# Sorption, solubility and cytotoxicity of novel antibacterial nanofilled dental adhesive resins

**DOI:** 10.1038/s41598-020-70487-z

**Published:** 2020-08-11

**Authors:** Fernando Luis Esteban Florez, Hannah Kraemer, Rochelle Denise Hiers, Catharina Marques Sacramento, Adam Justin Rondinone, Karina Gonzales Silvério, Sharukh S. Khajotia

**Affiliations:** 1 Division of Dental Biomaterials, Department of Restorative Sciences, College of Dentistry, The University of Oklahoma Health Sciences Center, 1201 North Stonewall Avenue, Oklahoma City, OK 73117 USA; 2 Department of Physiological Sciences, Piracicaba Dental School, State University of Campinas, Av. Limeira, 901-Bairro Areião, Piracicaba, São Paulo 13414-903 Brazil; 3 Department of Prosthodontics and Periodontics, Piracicaba Dental School, State University of Campinas, Av. Limeira, 901-Bairro Areião, Piracicaba, São Paulo 13414-903 Brazil; 4 Oak Ridge National Laboratory, Center for Nanophase Materials Sciences, Oak Ridge, TN 37831 USA

**Keywords:** Health care, Dentistry, Dental materials, Dental biomaterials

## Abstract

Dental adhesives hydrolyze in the mouth. This study investigated the water sorption (SOR), solubility (SOL) and cytotoxicity (CYTO) of experimental adhesives containing nitrogen-doped titanium dioxide nanoparticles (N_TiO_2_). Specimens (n = 15/group [SOR, SOL]; n = 10/group [CYTO]) of unaltered Clearfil SE Protect (CSP), OptiBond Solo Plus (OSP), Adper Scotchbond (ASB) and experimental adhesives (OSP + 25% or 30% of N_TiO_2_) were fabricated, desiccated (37 °C) and tested for SOR and SOL according to ISO Specification 4049 (2009). CYTO specimens were UV-sterilized (8 J/cm^2^) and monomer extracted in growth medium (1, 3 or 7 days). Human pulp cells were isolated and seeded (0.5 × 10^4^) for MTT assay. SOR and SOL data was analyzed using GLM and SNK (α = 0.05) and CYTO data was analyzed with Kruskal–Wallis and SNK tests (α = 0.05). SOR and SOL values ranged from 25.80 μg/mm^3^ (30% N_TiO_2_) to 28.01 μg/mm^3^ (OSP) and 23.88 μg/mm^3^ (30% N_TiO_2_) to 25.39 μg/mm^3^ (25% N_TiO_2_). CYTO results indicated that pulp cells exposed to experimental materials displayed comparable viabilities (p > 0.05) to those of OSP. Experimental materials displayed comparable SOR, SOL and CYTO values (p > 0.05) when compared to unaltered materials. N_TiO_2_ incorporation have not adversely impacted SOR, SOL and CYTO properties of unaltered adhesives.

## Introduction

Resin composite restorations are currently the most prevalent medical intervention in human beings with more than five hundred million restorations placed globally every year^1^. Such popularity amongst patients and clinicians precipitates from their mercury-free compositions^2^, outstanding esthetic properties, and their minimally invasive and ultra-conservative restorative techniques^3^. Their clinical use involves the removal of demineralized and bacteria-contaminated tooth structure, application of phosphoric acid (37%, 15–30 s), and the subsequent application of a primer and a dental adhesive resin in preparation for the intraoral fabrication of the resin composite restoration^4^.

The formation of the hybrid layer starts with the penetration of uncured monomers into water-rich, mineral-depleted areas of dentin, followed by the envelopment of exposed collagen fibrils, and the subsequent in situ polymerization by on-demand visible light irradiation^5^. Its successful completion^6^ should allow for the establishment of a crosslinked^7^ and hermetically sealed 3-dimensional polymer-collagen network^6^ capable of reducing microleakage, bacterial invasion, marginal staining, secondary caries and pulpal irritation^8^. However, because these materials contain both hydrophilic and hydrophobic moieties in a single product^9^, they tend to become chemically unstable when placed in contact with moist dentin^10^. The physical manifestation of such instability is translated into materials that phase-separate^11^ and display inadequate degrees of conversion^12^.

These problems are exacerbated further when trying to bond to dentin, because its water content, mineral composition and dentinal tubule morphology significantly change in a depth-dependent manner, where deep regions of dentin, are characterized by larger diameter tubules and a constant flow of dentinal fluids^13^. Therefore, this naturally wet, highly-heterogeneous and challenging substrate hinders the ability of current dental adhesive resins to adequately seal the margins of composite restorations^14,15^. The combination of the factors cited results in the formation of a porous and failure-prone adhesive layer that is highly susceptible to degradation by saliva, salivary enzymes (such as esterases) and biofilms. The clinical manifestation of these factors are materials with short service lives (5.7 years)^16^, higher incidences of secondary caries and higher oral health-care costs^17^. In addition, the utilization of hydrophilic monomers in dental adhesives has been shown to adversely affect the water sorption and solubility properties of these types of dental biomaterials^18–21^, and to result in the attainment of a semi-permeable hybrid layer susceptible to slow processes of hydrolytic degradation. Such high permeability facilitates the absorption of water (via diffusion^9^ and capillarity^20^), promotes the formation of water trees (a 3-dimensional network of interconnecting pores), and results in polymeric matrixes that are expanded and plasticized, and the leaching of unreacted hydrophilic monomers (such as TEGDMA, HEMA, bis-GMA, UDMA, etc.).

Such findings are very concerning from a biocompatibility standpoint, because those monomers have been correlated with concentration- and time-dependent cytotoxic and cell-modulating properties^10,22^ that may adversely impact basic cell functions including proliferation, mitochondrial respiration, homeostasis, cell morphology and the activity of intra-cellular enzymes^23^. Studies investigating the impact of viscosity, degree of monomer conversion and hydrophilicity on the cytotoxicity properties of dental adhesive resins against human pulp cells^24^, reported that cytotoxicity is directly correlated to the solubility of components present in dental adhesive resins. Other reports have also indicated that hydrophilic monomers are capable of upregulating the aggregation and growth of pathogenic biofilms^25,26^.

Previous studies have shown that titanium dioxide nanoparticles (n-TiO_2_) may be successfully incorporated into commercially available polymer compositions as a promising strategy to improve the properties (antibacterial and bioactivity) of dental adhesive resins^18,27^. These metaloxide nanoparticles display excellent mechanical properties (high elastic modulus and hardness), are hydrolysis resistant, cost-effective and are capable of producing reactive oxygen species (ROS) when irradiated with UV. More recently, a study investigated the antibacterial and bioactive properties of visible light-responsive nitrogen-doped titanium dioxide nanoparticles (N_TiO_2_) immobilized in a commercially available dental adhesive resin (OptiBond Solo Plus; Kerr, Orange, USA)^28^. Nevertheless, despite the promising properties previously reported^28^, no information is available regarding the sorption, solubility and cytotoxicity behavior of experimental dental adhesive resins containing N_TiO_2_.

Therefore, the objective of the present study was to investigate the impact of the incorporation of 25% and 30% (v/v) of N_TiO_2_ on the water sorption, solubility and cytotoxicity properties of OptiBond Solo Plus. The rationale for selecting 25% and 30% concentrations of N_TiO_2_ was based on a previous study^28^ wherein strong and promising antibacterial and bioactive properties were demonstrated in both dark and light-irradiated conditions at those concentrations. The testing of a commercial adhesive resin containing either 25% or 30% (v/v) of N_TiO_2_ is expected to uphold the null hypothesis tested that nanoparticles incorporation would not adversely impact the sorption, solubility and cytotoxicity properties of OptiBond Solo Plus. The results reported in the present study are anticipated to expand our current knowledge regarding how nanotechnology can be used to improve the service lives of methacrylate-based dental restorations.

## Materials and methods

### Synthesis of nanoparticles

The detailed description of the synthesis of N_TiO_2_ used in the present study has been previously reported by our laboratory^28^. In brief, nanoparticles were synthesized in two steps using very robust and controllable solvothermal reactions^29,30^. In the first step a solution composed of 1.7 g of Ti(IV)-butoxide 97% (Aldrich, St. Louis, USA), 4.6 g ethanol 200-proof (Decon Labs, King of Prussia, PA, USA), 6.8 g oleylamine 70% (Aldrich, St. Louis, MO, USA), 7.1 g oleic acid 90% (Aldrich, St. Louis, MO, USA) was prepared and mixed with ethanol (4% water; 20 mL, 18-Milli-Q). Both solutions were transparent before mixing, however, the final solution was clouded due to hydrolysis and formation of micelles. Aliquots (20 mL/each) of the final mixture were then individually placed into separate Teflon-lined high-pressure reaction vessels (Paar Series 5000; Moline, IL, USA), reacted at 180 °C (24 h) and stirred via external magnetic field. Room-temperature solutions were decanted and washed 3 times using 200-proof ethanol (Decon Labs, King of Prussia, PA, USA) to render pure TiO_2_ nanoparticles (n-TiO_2_). A portion of n-TiO_2_ suspended in ethanol were then reacted (at 140 °C) with an equal volume of triethylamine 99.5% (Aldrich, St. Louis, MO, USA) for 12 h as previously described. The now nitrogen-doped titanium dioxide nanoparticles (N_TiO_2_) were then washed 3 times with 200-proof ethanol and the concentration of particles was gravimetrically determined to be approximately 40 mg/mL.

### Dental adhesive resins

Three commercially available dental adhesive resins Clearfil SE Protect (CSP; Kuraray Noritake Dental, Tokyo, Japan), OptiBond Solo Plus (OSP; Kerr, Orange, CA, USA), Adper Scotchbond (ASB; 3 M ESPE, St. Paul, MN, USA) and two experimental dental adhesives (OSP + 25% or 30% of N_TiO_2_, [v/v]) were tested for SOR and SOL according to the ISO Specification 4049 (2009)^31^ with the exception of specimens’ dimensions and immersion time that were modified according to a previously published protocol^9^. Experimental adhesive resins were prepared in a similar manner to previous studies^32,33^. Specimens (n = 15; diameter = 6.0 mm; thickness = 0.5 mm) of OSP, 25% N_TiO_2_, and 30% N_TiO_2_ were manually fabricated by individually pouring each dental adhesive resin into the wells of a custom-made metallic mold. Each well was then polymerized (from the top) for 60 s using an LED light curing unit (VALO; Ultradent Products, South Jordan, UT, USA) following a protocol previously reported^28^. Specimens of CSP and ASB (n = 15; diameter = 6.0 mm; thickness = 0.5 mm) were fabricated using custom-made silicone molds (Reprosil; Dentsply Caulk, Milford, DE, USA). Each specimen was then individually polymerized using the same method described previously.

### Water sorption and solubility

Following fabrication, specimens in each group were identified, placed in petri dishes, and stored in a desiccator containing fresh silica gel packs. The desiccator was then placed in an incubator at 37 °C. Specimens were weighed every 24 h using an analytical scale (Mettler-Toledo, Columbus, OH, USA) and the diameter and thickness were measured using a digital caliper until a constant mass (*m*_1_) was achieved. In the present study constant mass was defined as mass variations less than 0.2 mg within a 24-h interval.

After specimens reached a constant mass, each specimen was individually placed into separate test tubes containing 10 mL of sterile ultrapure water (pH 7.2) at oral temperature (37 °C). The specimens were then stored in an incubator (at 37 °C) for 1, 2, 3, 4, 5, 6, 7, 15 and 30 days. After each storage time, specimens were removed from the incubator and allowed to sit at room temperature for 30 min. Specimens were then removed from their test tubes and washed in a sonicated water-bath for one minute at room temperature using sterile ultra-pure water (pH 7.2). After that, specimens were blotted dry (Kimwipes; Kimtech Science, Milsons Point, NSW, Australia) and weighed (*m*_2_) with an analytical scale. After 30 days of water immersion, specimens were removed from test tubes and were placed in petri dishes. Specimens were then stored in a desiccator containing fresh silica gel packs. The desiccator containing the petri dishes with the specimens was then placed in an incubator (at 37 °C). In 24-h intervals, the specimens were weighed and measured until a constant mass (*m*_3_) was achieved. The 30-day water sorption (SOR) and solubility (SOL) values were then calculated according to ISO Specification 4049 (2009)^31^ using the following formulae:

1 $$SOR = \frac{{\left( {m_{2} - m_{3} } \right)}}{V}$$

2 $$SOL = \frac{{\left( {m_{1} - m_{3} } \right)}}{V}$$

### Cytotoxicity analysis

#### Extraction of dental adhesives eluates

An additional set of specimens (n = 10/group; diameter = 6.0 mm; thickness = 0.5 mm) was fabricated using both the unaltered (CSP, OSP, ASB) and experimental (OSP + 25% or 30% of N_TiO_2_, [v/v]) dental adhesive resins using the same protocol as previously described (session “Dental adhesives”). Specimens were then individually transferred into separate wells of sterile 24-well plates (Corning, New York, NY, USA) and were UV-sterilized (8 J/cm^2^, UVP CL-1000 Crosslinker; Upland, CA, USA). Aliquots (300 μL) of sterile Dubelcco’s Modified Eagle Medium (DMEM; Life Technologies, Carlsbad, CA, USA) supplemented with 10% fetal bovine serum (FBS; Life Technologies, Carlsbad, CA, USA) was added to each well containing the specimens. The 24-well plates containing the specimens and growth medium were incubated (37 °C) for the periods of 1, 3 and 7 days for the extraction of materials’ eluates. The surface area to volume ratio was 0.94 cm^2^/mL, which was set according to ISO Specification 10993 (0.5–6.0 cm^2^/mL) and previous publications^34,35^. At the end of each incubation period of time, the supernatant was carefully collected using calibrated pipettes and were stored (dark conditions) in sterile test tubes at − 20 °C until further use.

#### Cell culture

The present study was reviewed and approved by the Institutional Review Board of the Piracicaba Dental School, University of Campinas (# 3.804.732). The international ethical guidelines for biomedical research involving human subjects^36^ were followed in the present study. Three human pulp cell populations were isolated and maintained by the Periocells Biobank at the Cellular and Molecular Biology Laboratory of the Piracicaba Dental School as previously described^37^. Pulp cells were cultured in standard medium composed of DMEM supplemented with 10% FBS and 1% penicillin/streptomycin (P/S; Life Technologies, Carlsbad, CA, USA) at 37 °C in atmosphere containing 5% CO_2_. Populations of pulp cells from the second to the fourth passage were used in triplicate in the present study.

#### Cell viability assay

The viability of cells incubated in materials’ eluates was determined by using 3-[4,5-dimethylthiazol-2yl]-2,5-diphenyl tetrazolium bromide assay (MTT; Sigma-Aldrich, St. Louis, MO, USA). Cells were seeded into separate wells of 96-well plates (0.5 × 10^4^ cells/well) in standard medium for 24 h for cell attachment and spreading. Subsequently, cells were replenished using fresh medium (DMEM + 10% FBS and 1% P/S) with or without materials’ eluates (either 20%, 40% or 80%)^35^ and were incubated (37 °C, 72 h). The MTT reagent was then added to individual wells and cells were incubated (37 °C, 4 h) in a humidified 5% CO_2_ incubator. At the end of the incubation period, spent medium was carefully aspirated and converted dye was solubilized using 200-proof ethanol (Decon Labs, King of Prussia, PA, USA). The absorbance of the formazan dye formed was then measured photometrically at 570 nm (VersaMax ELISA Microplate Reader; Molecular Devices, San Jose, CA, USA). Cell cultures that were not exposed to materials’ eluates served as the negative control group (100% viability).

### Statistical analysis

Data obtained for *SOR* and *SOL* were statistically analyzed using General Linear Models (GLM) and Student Newman Keuls (SNK) post hoc tests. Data for CYTO was assessed for normality using the Shapiro–Wilk test. Since data was not normally distributed, the non-parametric Kruskal–Wallis and the Student–Newman–Keuls post hoc tests (α = 0.05) were then used when statistically significant differences among experimental groups were identified. Statistical analyses for SOR and SOL and CYTO were performed using SAS software (Version 9.2; SAS Institute, Cary, NC, USA) and for CYTO using Bioestat software (Version 5.3; Sociedade Civil de Mamirauá, Belém, PA, Brazil), respectively.

## Results

**Figure 1:**
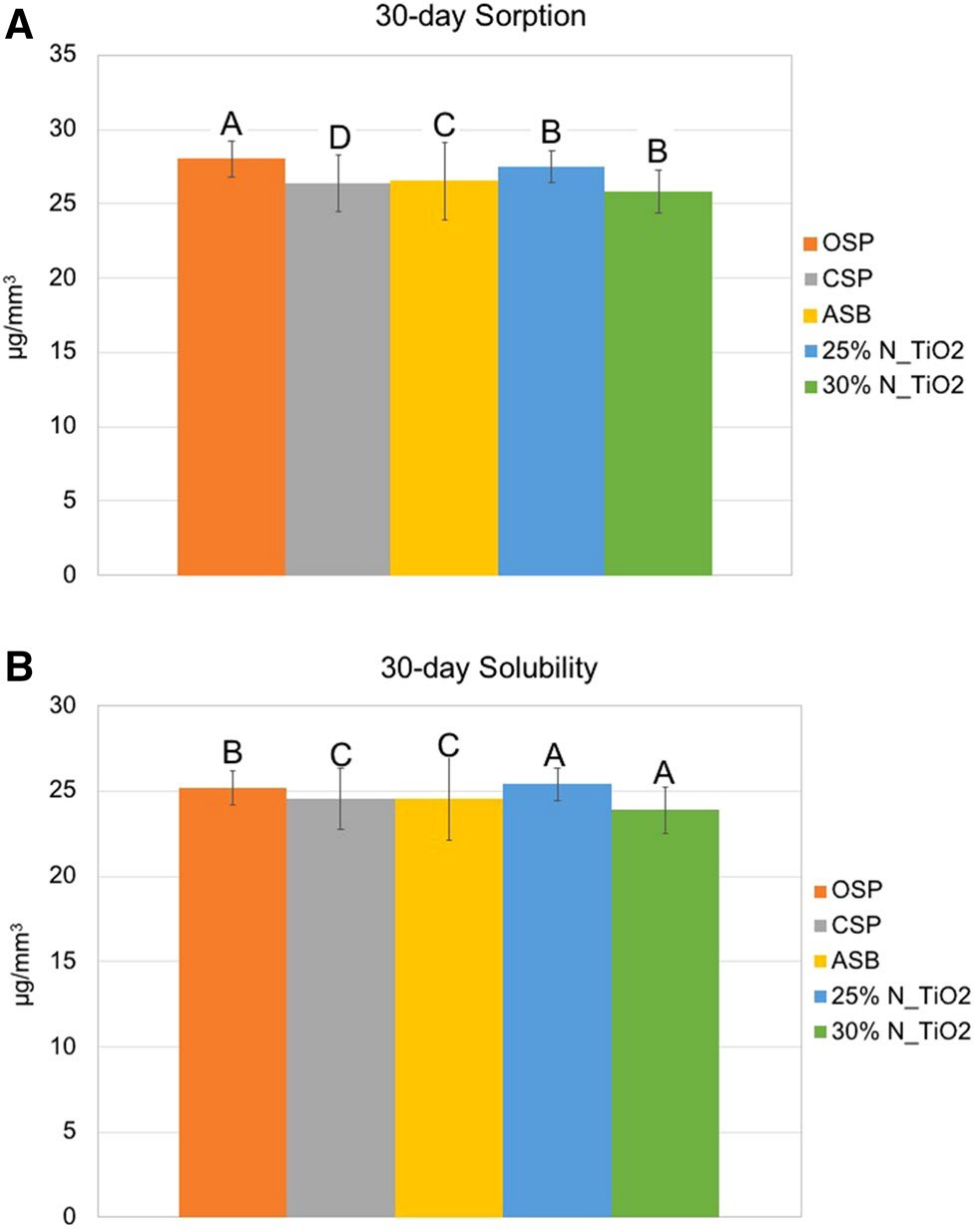
Mean and standard deviation values of 30-day (**A**) sorption and (**B**) solubility of unaltered (ASB, SBP, CSP and OSP) and experimental (OSP + either 25% or 30% of N_TiO_2_, [v/v]).

Figure 1A,B illustrate the mean and standard deviation values of the 30-day SOR and SOL tests of specimens fabricated with both unaltered (ASB, CSP and OSP) and experimental (25% N_TiO_2_ and 30% N_TiO_2_) dental adhesive resins. Figure 1A shows the results of the 30-day SOR test, where mean values ranged from 25.80 μg/mm^3^ (30% N_TiO_2_) to 28.01 μg/mm^3^ (OSP), and specimens fabricated with experimental adhesive resins containing either 25% or 30% of N_TiO_2_ displayed mean values of 30-day SOR (27.51 and 25.80 μg/mm^3^, respectively) that were lower as compared to those of the parental polymer OSP (28.01 μg/mm^3^). Figure 1B demonstrated that adhesive resins pertaining to experimental groups ASB, CSP, OSP, 25% N_TiO_2_ and 30% N_TiO_2_ displayed mean values of 30-day SOL that ranged from 23.88 μg/mm^3^ (30% N_TiO_2_) to 25.39 μg/mm^3^ (25% N_TiO_2_). Specimens fabricated with N_TiO_2_ (either 25 or 30% [v/v]) exhibited mean values of 30-day SOL that were comparable to those of specimens fabricated with unaltered ASB, CSP and OSP.

**Figure 2:**
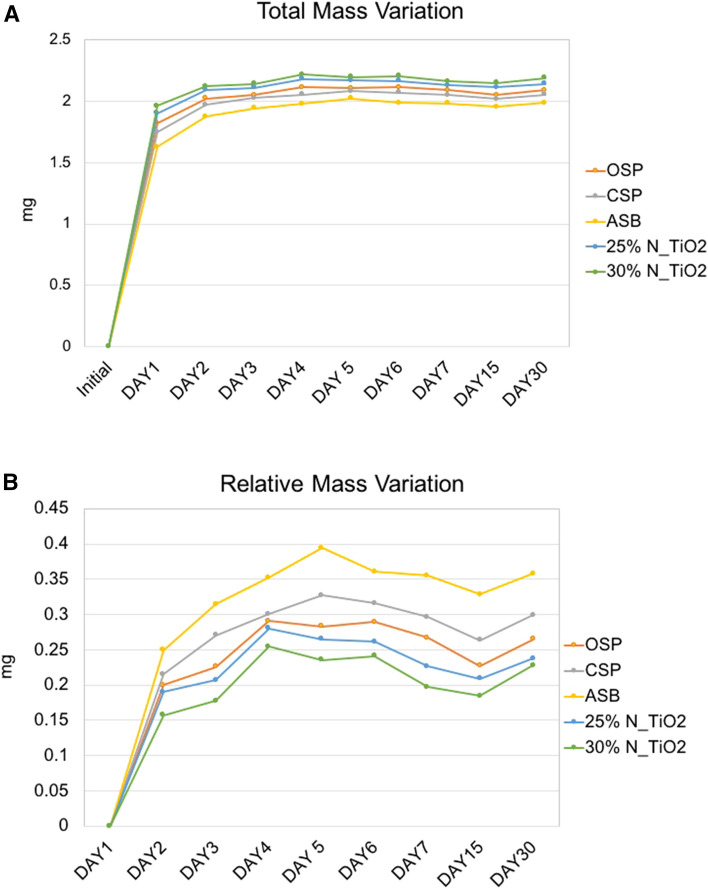
Mean values of (**A**) total mass variation (specimens’ weights + weight of water gained in each day) and (**B**) relative mass variation (water gained in each day).

Figure 2A illustrates the temporal evolution of total mass variation (initial specimen weight + water gain) and Fig. 2B shows the relative mass variation (water gain only). The highest variations in total mass were observed between days 1 and 5 independently of the type of material considered (either unaltered or experimental). At day 1 (after water immersion) specimens could be rank ordered in terms of decreasing values of weight where 30% N_TiO_2_ > 25% N_TiO_2_ > OSP > CSP > ASB, respectively. Specimens’ weights continually increased up until the fifth day. After that period of time (between days 6–30), specimens reached a plateau and no significant weight changes could be perceived. It can also be seen that the weight distribution trend observed at day 1 was maintained throughout the course of the 30-day immersion period, which could be an indication that experimental materials could have absorbed more water. However, when analyzing the results from the relative mass variation (Fig. 2B), it becomes clear that specimens fabricated with experimental materials containing either 25% or 30% of N_TiO_2_ actually displayed the lowest variations in relative mass due to water absorption among all groups investigated (30% N_TiO_2_ < 25% N_TiO_2_ < OSP < CSP < ASB), which indicates that nanoparticles’ incorporation may have improved the sorption and solubility behavior of OSP.

**Figure 3:**
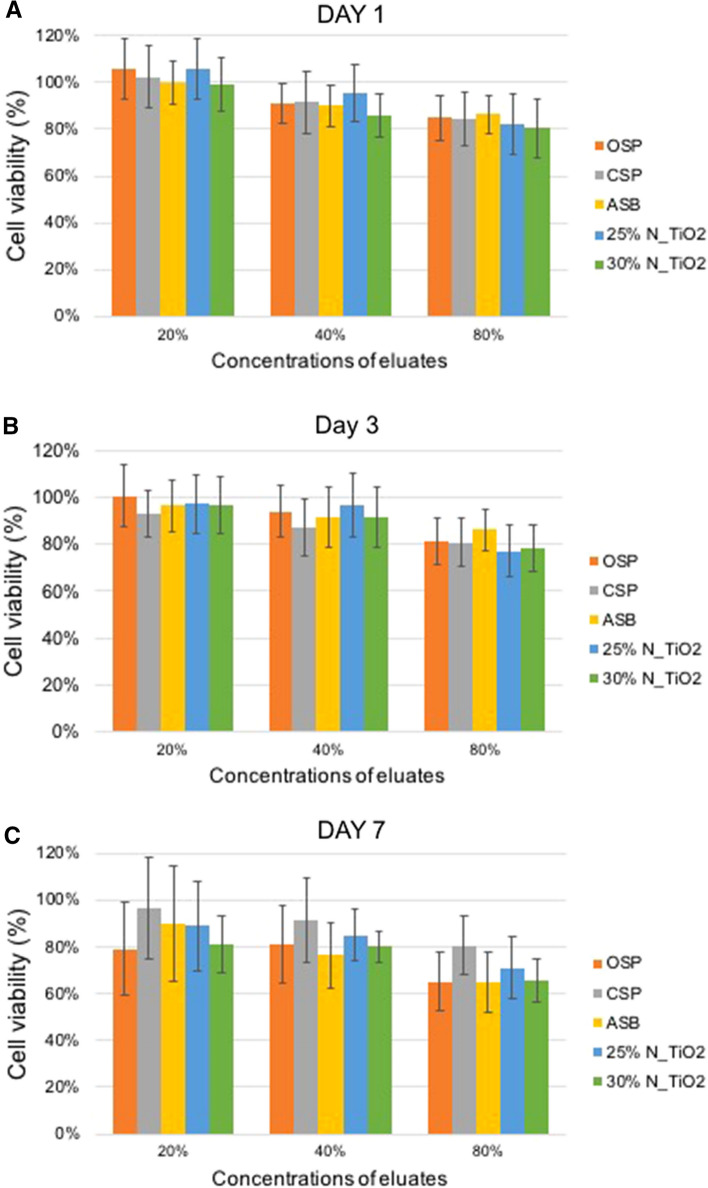
Viability of human pulp cells after being exposed to eluates (either 20%, 40% or 80%) of unaltered (ASB, SBP, CSP and OSP) and experimental (OSP + either 25% or 30% of N_TiO_2_, [v/v]) dental adhesive resins for (**A**) 1 day, (**B**) 3 days and (**C**) 7 days.

Figure 3A–C illustrates the results of the MTT viability assay of human pulp cells exposed to materials’ eluates (1, 3 or 7 days) in varying concentrations (20%, 40% and 80%). Figure 3A illustrates that cells exposed to growth medium containing 1-day eluates (20%) demonstrated viability levels that were comparable (p > 0.05) to those of the negative control group cells (DMEM only, 100% viability) independently of the material considered (ASB, CSP, OSP, 25% N_TiO_2_ or 30% N_TiO_2_). Cells exposed to standard growth medium containing higher concentrations of eluates (either 40% or 80%, independently of day [either 1, 3 and 7]), displayed viability levels that were significantly lower (p < 0.05) when compared to the viability levels of cells in the negative control group (100% viability). It is also possible to observe that an inverse relationship was established between the viability of cells and eluates’ concentration, where higher concentrations of eluates resulted in lower levels of cell viability, independently of material (unaltered or experimental) or eluate time (1, 3 or 7 days). Despite these results, no statistically significant differences (p > 0.05) were observed amongst experimental materials (containing either 25% or 30% of N_TiO_2_) and the parental polymer (FDA-approved), thereby suggesting that nanoparticles incorporation did not adversely impact the cytotoxicity of OSP.

## Discussion

The SOR and SOL properties of dental adhesive resins have been shown to directly impact the long-term success^15,38^ of esthetic restorative restorations. Dental adhesive resins are polymer-based biomaterials that are typically used in restorative dentistry to establish the bond between the tooth structure and resin composites. Typical limitations associated with current dental adhesive resins include polymerization shrinkage, incomplete enveloping of collagen fibrils, microleakage^39^ and up-regulation of dental biofilms^40^. These factors combined have been demonstrated to result in esthetic restorations displaying premature failure by secondary caries^41^, and shorter service lives when compared to dental amalgams and other restorative materials^16^.

The results reported in the present study have indicated that 30-day mean values of SOR and SOL ranged from 25.80 μg/mm^3^ (30% N_TiO_2_) to 25.39 μg/mm^3^ (25% N_TiO_2_) and 23.88 μg/mm^3^ (30% N_TiO_2_) to 28.01 μg/mm^3^ (OSP), respectively. These results have been corroborated by the findings of a previous study^9^ that investigated the SOR and SOL properties of four commercial dental adhesive resins according to ISO Specification 4049 (2009)^31^. In that study, the authors have indicated that SOR and SOL properties are strongly and positively correlated to materials’ compositions, hydrophilicity and polymerization kinetics^9^. In addition, results reported have demonstrated that SOR and SOL are inversely correlated to materials’ degrees of conversion, wherein highly crosslinked polymers will experience smaller amounts of SOR and SOL due to the formation of a dense polymer network with the presence of few unreacted hydrophilic monomer molecules^9^. Based on this physico-chemical principle, it is possible to infer that modern resin-based materials displaying low degrees of conversion, will be less biocompatible, and will experience high levels of SOR and SOL, lower mechanical properties and shorter service lives.

The results presented in Fig. 1A,B) indicate that the incorporation of nanoparticles (either 25% or 30% of N_TiO_2_, v/v) did not adversely impact SOR and SOL properties of OSP, as denoted by mean values that were not statistically different (p > 0.05) than those observed for unaltered OSP. Figure 2A has shown that specimens fabricated with experimental materials containing either 25% or 30% (v/v) of N_TiO_2_ were heavier (between 1 and 30 days) when compared to unaltered materials. This was an expected behavior that precipitated from the incorporation of large amounts of metaloxide nanoparticles. Such incorporation not only altered the specific gravity (higher mass per unit volume) of experimental materials, but also decreased the ability of these materials to absorb water, as shown (Fig. 2B) by experimental materials associated with the lowest values of relative mass variation among all materials investigated. This finding can be fully explained by the intrinsic properties of nanoparticles investigated, which are hydrolysis-resistant and do not absorb water. A study^27^ investigating the impact of thermocycling on the functionalization of n-TiO_2_ in dental resins has indicated that functionalized n-TiO_2_ become irreversibly entrapped within the polymer matrix due to the establishment of hydrogen bonds between oxides (OH) present on nanoparticles’ surfaces and organic components of the polymeric matrix.

The results reported in the present study, along with the body of literature available, have indicated that experimental nanofilled dental adhesive resins may be able to withstand the harsh conditions in the oral cavity, where dental biomaterials are continuously challenged in regards to temperature, pH, biofilm formation and cyclical masticatory forces. These factors combined, tend to degrade the properties (e.g., surface, mechanical and biological properties) of dental polymers and result in dental restorations with shorter service lives^42^. Several groups have tried to overcome current technological limitations by adding antibacterial monomers (e.g., quaternary ammonium dimethacrylates) and agents (e.g., chlorhexidine and antibiotics) or nanoparticles (e.g., Ag, ZnO and n-TiO_2_) into dental adhesive resins to improve their mechanical and surface properties (e.g., elastic modulus and hardness, respectively) while eliciting promising antibacterial effects when irradiated with UV-wavelengths^27,43^. In this direction, the addition of n-TiO_2_ nanoparticles into glass ionomer cements has been demonstrated to increase both the flexural and compressive strengths, as well as the fracture toughness of experimental materials investigated^44^. Another study^27^ demonstrated that adhesive resins containing nanoparticles (functionalized either by acrylic acid^45^ or acetic acid^46^) displayed values of degree of conversion, Knoop hardness, elastic moduli and shear bond strength that were higher and statistically significant (p < 0.05) when compared to the values of the unaltered materials investigated.

A recent study^28^ reported a simple and robust method to synthesize and incorporate visible light-responsive N_TiO_2_ in OSP. In that study, our group characterized the properties of nanoparticles (e.g., shape, size, composition and morphology, etc.) and experimental materials (e.g., antibacterial, bioactive, biaxial flexure strength, etc.) containing varying concentrations (5–80%, [v/v]) of functionalized N_TiO_2_. The results reported indicated that experimental materials synthesized displayed promising antibacterial properties against *Streptococcus mutans* biofilms (24-h) in both dark and light irradiated conditions^28^. The impact of different light-curing units (QTH or LED) on the SOR, SOL (water and ethanol) and residual monomers’ leachability of Adper Single Bond 2 (3 M ESPE, St. Paul, MN, USA) has been demonstrated previously^21^. According to results reported, the type of light source used directly influenced properties investigated where materials polymerized by an LED-source were observed to display lower levels of crosslinking and higher levels of SOR, SOL and monomer leaching^21^, which is a factor of fundamental importance for the biocompatibility of dental adhesive resins.

The present study’s rationale for the utilization of primary cells for cytotoxicity testing was based on a recent systematic review of the literature^4^, where it was suggested that non-immortalized and genetically intact human pulp cells are the most indicated study model to determine the cytotoxicity of dental adhesive resins^47^ and because these types of cells maintain tissue’s original characteristics. The results shown in Fig. 3A–C have clearly indicated that the incorporation of N_TiO_2_ in the concentrations investigated (either 25% or 30%, v/v) have not altered the cytotoxicity behavior of OSP, as denoted by comparable levels of cells’ viability. The results of the present study have been corroborated by a previous study^48^ that investigated the photocatalytic-induced bioactivity of experimental dental polymers containing varying concentrations of TiO_2_ nanoparticles. That study has shown that experimental nanocomposites investigated were nontoxic against two human cell lines (human dermal fibroblasts [hDF] and osteoblast-like human sarcoma [MG63]) in both dark and UV-irradiated conditions^48^. The results reported in the present study have allowed us to not reject the null hypothesis tested that the incorporation of N_TiO_2_ (25% and 30%, v/v) did not adversely impacted the sorption, solubility and cytotoxicity properties of OSP, thereby providing further scientific evidence to support the addition of N_TiO_2_ into commercially available dental adhesive resins as additives to improve relevant properties (physical, chemical and biological) of current dental polymers.

Future studies in this field should investigate the effects of nanoparticles that are surface-modified with silanes and proteins on the SOR, SOL and CYTO of experimental dental adhesives. Moreover, studies regarding the mechanisms by which doped- or co-doped TiO_2_ nanoparticles improve physical, mechanical and biological properties of dental adhesive resins should be performed to expand our current understanding regarding the positive impacts of nanotechnology in dental biomaterials.

## Conclusions

The present study has demonstrated the successful preparation of antibacterial and bioactive N_TiO_2_ nanoparticles using robust and controllable solvothermal reactions, their incorporation into current dental adhesive resins and the impacts of nanoparticles’ incorporation on the sorption, solubility and cytotoxicity characteristics of OptiBond Solo Plus, which is a commercially available dental adhesive resin. Experimental materials containing either 25% or 30% (v/v) of N_TiO_2_ were shown to display comparable water sorption and solubility properties to those of unaltered OSP, CSP and ASB. Cytotoxicity results indicated that experimental materials containing either 25% or 30% (v/v) of N_TiO_2_ were as biocompatible as commercially-available adhesive resins, thereby strongly supporting the safe utilization of metaloxide nanotechnology in modern dentistry.
